# Phenotypic and genotypic characterization of Thai isolates of *Plasmodium falciparum* after an artemisinin resistance containment project

**DOI:** 10.1186/s12936-018-2347-9

**Published:** 2018-05-15

**Authors:** Thunyapit Thita, Pimrat Jadsri, Jarupatr Thamkhantho, Toon Ruang-areerate, Nantana Suwandittakul, Naruemon Sitthichot, Kittiya Mahotorn, Peerapan Tan-ariya, Mathirut Mungthin

**Affiliations:** 1 Department of Microbiology, Faculty of Science, Mahidol University, Bangkok, Thailand; 2 Department of Parasitology, Phramongkutklao College of Medicine, Bangkok, Thailand

**Keywords:** *Plasmodium falciparum*, Drug resistance, Thai-Cambodian, In vitro sensitivity, Genetic markers, Artemisin-based combination therapy, Artemisinin resistance containment project

## Abstract

**Background:**

In Thailand, artemisinin-based combination therapy (ACT) has been used to treat uncomplicated falciparum malaria since 1995. Unfortunately, artemisinin resistance has been reported from Thailand and other Southeast Asian countries since 2003. Malarone^®^, a combination of atovaquone–proguanil (ATQ–PG), has been used to cease artemisinin pressure in some areas along Thai–Cambodia border, as part of an artemisinin resistance containment project since 2009. This study aimed to determine genotypes and phenotypes of *Plasmodium falciparum* isolates collected from the Thai–Cambodia border after the artemisinin resistance containment project compared with those collected before.

**Results:**

One hundred and nine of *P. falciparum* isolates collected from Thai–Cambodia border from Chanthaburi and Trat provinces during 1988–2016 were used in this study. Of these, 58 isolates were collected after the containment. These parasite isolates were characterized for in vitro antimalarial sensitivities including chloroquine (CQ), quinine (QN), mefloquine (MQ), piperaquine (PPQ), artesunate (AS), dihydroartemisinin (DHA), ATQ and PG and genetic markers for drug resistance including the Kelch13 (*k13*), *Plasmodium falciparum chloroquine resistance transporter* (*pfcrt*), *P. falciparum multidrug resistance 1* (*pfmdr1*) and *cytochrome b* (*cytb*) genes. Mean CQ, QN, MQ, PPQ and AS IC_50_s of the parasite isolates collected from 2009 to 2016 exhibited significantly higher than those of parasites collected before 2009. Approximately 57% exhibited in vitro MQ resistance. Approximately 94% of the isolates collected from 2009 to 2016 contained the *pfmdr1* 184F allele. Mutations of the *k13* gene were detected in approximately 90% of the parasites collected from 2009 to 2016 which were significantly higher than the parasite isolates collected before. No ATQ-resistant genotype and phenotype of *P. falciparum* were found among the isolates collected after the containment project.

**Conclusions:**

Although the containment project had been implemented in this area, the expansion of artemisinin-resistant parasites did not decline. In addition, reduced sensitivity of the partner drugs of ACT including MQ and PPQ were identified.

## Background

Malaria is one of the important parasitic diseases threatening human beings over many decades. Emergence and spread of drug resistance is an important cause of morbidity and mortality in malaria. Artemisinin derivatives are the most potent drugs against multidrug-resistant *Plasmodium falciparum*. Artemisinin-based combination therapy (ACT) has been recommended by the World Health Organization (WHO) to use as the first-line treatment for multidrug-resistant falciparum malaria [1]. Artesunate-mefloquine (AS-MQ) had been used in Thailand to treat uncomplicated falciparum malaria since 1995 due to the emergence of MQ resistance [2]. Later AS–MQ was used in other Southeast Asian countries including Cambodia [2]. Unfortunately, the efficacy of AS–MQ declined along the Thai-Cambodian border after a few years of implementation [3, 4]. Increased AS–MQ failure rates observed in Thailand and Cambodia were usually associated with MQ resistance [5, 6]. Recently, a few studies have shown evidence of artemisinin resistance in *P. falciparum*, defined as delayed parasite clearance, i.e., presence of parasitaemia on day 3 following treatment with AS monotherapy or ACT [7, 8]. Subsequently, artemisinin resistance has emerged independently and spread to many areas of the Greater Mekong Subregion (GMS) [9, 10]. To determine the situation of artemisinin resistance, a novel ring survival assays (RSA) (i.e., in vitro or ex vivo RSA) has been used to represent the delayed parasite clearance phenotype [11]. Ariey et al. have identified mutations of the Kelch13 (*k13*) gene as molecular markers for artemisinin resistance [12]. Mutations in the *k13* gene were correlated with delayed parasite clearance and also increased RSA (0–3 h) rate [12, 13]. The mutations were associated with artemisinin resistance in Cambodia and other countries in the GMS [12, 14–16].

Due to the emergence of artemisinin resistance in some areas along the Thai-Cambodian border, an artemisinin resistance containment project has been launched to cease the spread of artemisinin resistance since 2009 [17]. The combination of AS–MQ has been replaced by a fixed-dose combination of atovaquone–proguanil (ATQ–PG) (Malarone^®^) to reduce artemisinin pressure in these areas. ATQ inhibits the mitochondrial electron transport chain at the bc1 complex [18, 19]. Mutations in the *cytochrome b* (*cytb*) gene resulted in amino acid changes at codon 268, exchanging tyrosine for serine (Y268S) or, less frequently, asparagine (Y268 N) conferred ATQ resistance and treatment failure [20, 21]. Monitoring in vitro drug sensitivities and also molecular markers of *P. falciparum* isolates is essential to detect the emergence and spread of drug resistance and provide valuable information for a rational drug use policy. Little is known about the characteristics of *P. falciparum* after the artemisinin resistance containment project. This study was aimed to determine phenotypes and genotypes of *P. falciparum* isolated from the Thai–Cambodian border after the artemisinin resistance containment project compared with those collected before.

## Methods

### Parasite isolates and cultivation

One hundred and nine *P. falciparum* adapted isolates from along the Thai-Cambodia border of Chanthaburi and Trat Provinces from 1988 to 2016, were used in this study. The research protocol was reviewed and approved by the Ethics Committee of the Royal Thai Army Medical Department. Of these, 51 frozen adapted isolates were collected between 1988 and 2005 from Chanthaburi province. Thirty-six isolates was collected from 1988 to 1993, while fifteen isolates were collected between 2003 and 2005. Fifty-eight newly adapted isolates were collected from the border of Trat province from 2009 to 2016, when the artemisinin resistance containment project was implemented. No clinical profiles and outcomes of the patients were recorded. All isolates were cultured using the modification method of Trager and Jensen [22]. Parasite isolates were cultured in culture flasks containing medium RPMI 1640 (Gibco^®^), 10% human AB serum and human erythrocytes (O^+^). Culture flasks were maintained under an atmosphere of 90% N_2_, 5% O_2_, and 5% CO_2_ and incubated at 37 °C.

### In vitro drug sensitivity assay

Newly adapted isolates were usually maintained in the culture for 2–3 cycles before in vitro sensitivity assays. *Plasmodium falciparum* isolates collected before and after 2009 were randomly measured for the IC_50_ at the same time to minimize batch effects. In addition, the reference laboratory strains, K1 and 3D7 were used as a control in each batch of experiment. Drug sensitivities of *P. falciparum* isolates to chloroquine (CQ), quinine (QN), MQ, piperaquine (PPQ), AS, dihydroartemisinin (DHA), ATQ and PG were determined by measuring [^3^H] hypoxanthine incorporated in parasite nucleic acids using the modified technique of Desjadins et al. [23]. The GRAFIT^®^ Program (Erithacus Software Limited, UK) was used to determine inhibitory concentration 50% (IC_50_).

### Characterization of the *pfcrt, pfmdr1*, *cytb* and *k13* genes

*Plasmodium falciparum* DNA was extracted using the Chelex-resin method [24]. PCR–RFLP techniques were performed to detect *pfcrt* mutation at codon 76, *pfmdr1* mutation at codons 86, 184, 1034, 1042 and 1246 and *cytb* mutation at codon 268 as previously described [20, 25, 26]. The *pfmdr1* gene copy number was detected by TaqMan real-time PCR (CFX96™ Real-Time PCR Detection System; Bio-Rad Laboratories, Inc.) [27]. The K1 and DD2 clone containing 1 and 4 *pfmdr1* copies, respectively, were used as the reference DNA sample. The amplification of *pfmdr1* and *β*-*tubulin* was performed in triplicate and relative *pfmdr1* copy number was determined [27].

The *k13* gene was amplified using the method of Ariey et al. [12]. PCR products were performed and visualized using 2% agarose gel electrophoresis and SYBR^®^ Safe DNA Gel Stain (Thermo Fisher Scientific). The PCR products were purified using the QIAquick^®^ Gel Extraction Kit and sequenced by U2Bio Co. Ltd. (Seoul, South Korea). The sequences were aligned against the *P. falciparum* 3D7 strain (PF3D7_1343700, PlasmoDB Release 28) using BioEdit v7.2.5.

### Statistical analysis

Data were analysed by STATA/MP, Version 12. Each IC_50_ value represented the mean of at least three independent experiments. Each in vitro drug sensitivity experiment was performed in triplicate. Normally distributed IC_50_ data were assessed by the Kolmogorov–Smirnov test. Differences of the mean IC_50_ and copy number of the *pfmdr1* gene among groups were analysed by Independent *t* test and One-way ANOVA. Post hoc test (Scheffe) for multiple comparisons was used to test for differences between the two groups. Associations between genotypes and *P. falciparum* from different areas were analysed by Chi square or Fisher’s exact test and level of significance was set at a *p* value of < 0.05.

## Results

### In vitro drug sensitivities of *Plasmodium falciparum*

In vitro drug sensitivities of *P. falciparum* isolates collected before and after 2009 are presented in Table 1. Mean CQ, QN, MQ, PPQ and AS IC_50_s of the parasite isolates collected from 2009 to 2016 showed significantly higher value than those of parasites collected before 2009. In contrast, mean DHA IC_50_s of the parasites before 2009 and 2009–2016 were not significantly different. In the present study, only ATQ and PG IC_50_s of the parasites collected from 2009 to 2016 were shown.

**Table 1:** In vitro drug sensitivity of *P. falciparum* isolated from along the Thai–Cambodia border collected before 2009 and from 2009 to 2016

Drug	Mean IC_50_ (n)	Min–Max IC_50_	Mean IC_50_ Before 2009 (n)	Mean IC_50_ 2009–2016 (n)	*p* value
CQ	105.0 ± 53.0 (101)	13.9–334.6	90.2 ± 37.5 (50)	120.5 ± 61.4 (51)	0.004
QN	218.4 ± 131.5 (101)	34.5–737.9	181.4 ± 107.9 (50)	256.5 ± 142.0 (51)	0.004
MQ	28.2 ± 25.8 (101)	1.7–130.9	20.4 ± 23.1 (50)	35.5 ± 26.1 (51)	0.003
PPQ	22.0 ± 11.9 (90)	6.4–74.8	17.1 ± 6.9 (43)	26.1 ± 13.6 (47)	< 0.001
ARS	3.0 ± 1.8 (101)	0.5–9.7	2.6 ± 1.3 (50)	3.5 ± 2.1 (51)	0.008
DHA	2.5 ± 1.2 (95)	0.8–6.2	2.6 ± 1.2 (44)	2.3 ± 1.1 (51)	0.218
ATQ	1.5 ± 1.3 (50)	0.2–6.7	–	1.5 ± 1.3 (50)	–
PG	53.9 ± 20.3 (50)	21.5–115.2	–	53.9 ± 20.3 (50)	–

Significant differences of drug IC_50s_ between parasites collected before 2009 and from 2009 to 2016 determined using Independent *t* test

Table 2 shows the percentage of resistant phenotypes of *P. falciparum* isolated along the Thai–Cambodian border in different years. Cut-off points for in vitro anti-malarial resistance were used as previously described [28–32]. In vitro CQ, MQ and QN resistance were defined when the IC_50_ ≥ 25 nM, > 30 nM and > 800 nM, respectively [28–30]. However, according to Pradines et al., AS and DHA resistance have been categorized when the IC_50_ was more than 10.5 nM [31]. In vitro ATQ resistance was defined when the parasites exhibited the IC_50_ of more than 1900 nM [32]. The cut-off point of PPQ resistance has not been defined yet. Nearly all parasite isolates exhibited in vitro CQ resistance. Parasite isolates collected in 2009 contained significantly higher MQ-resistant phenotype (56.9%) than the parasites before 2009 (22.0%) (*p *< 0.001, Fisher’s Exact test). No parasite isolates showing QN, AS, DHA or ATQ resistance were identified.

**Table 2:** The percentage of resistant phenotypes of *P. falciparum* isolated from along the Thai–Cambodia border collected before 2009 and from 2009 to2016

Drug	Total% (n)	Before 2009% (n)	2009–2016% (n)	*p* value
CQ	99.0 (100/101)	98.0 (49/50)	100 (51/51)	0.500
QN	0 (0/101)	0 (0/50)	0 (0/51)	–
MQ	39.6 (40/101)	22.0 (11/50)	56.9 (29/51)	< 0.001
ARS	0 (0/101)	0 (0/50)	0 (0/51)	–
DHA	0 (0/95)	0 (0/44)	0 (0/51)	–
ATQ	0 (0/50)	–	0 (0/50)	–

Significant differences of resistant phenotype between parasites collected before 2009 and from 2009 to 2016 determined using Fisher’s Exact test

CQ resistance was defined as IC_50_ ≥ 25 nM, QN resistance was defined as IC_50_ > 800 nM, MQ resistance was defined as IC_50_ > 30 nM, ARS resistance was defined as IC_50_ > 10.5 nM, DHA resistance was defined as IC_50_ > 10.5 nM and ATQ resistance was defined as IC_50_ > 1900 nM

### Polymorphisms of *pfcrt, pfmdr1*, *cytb* and *k13* genes

*Plasmodium falciparum* isolates collected in the years before and after the artemisinin resistance containment project from Chanthaburi and Trat Provinces were characterized for the polymorphisms of the *pfcrt*, *pfmdr1*, *cytb* and *k13* genes (Table 3). Of 104 isolates, only two contained the wild-type *pfcrt* gene. Approximately 89% of these parasites had the *pfmdr1* 184F allele. The copy number of *pfmdr1* had approximately 1 copy number (1.1 ± 0.6, n = 102) as shown in Table 3. The wild-type *cytb* gene at the codon 268 was identified in all parasite isolates both before and after the artemisinin containment project.

**Table 3:** Resistant gene polymorphisms of the parasite isolates collected before 2009 and from 2009 to 2016

	Total	Before 2009	2009–2016	*p* value
*pfcrt* 76T, % (n)	98.1 (102/104)	96.0 (48/50)	100 (54/54)	0.229
*pfmdr1*				
Copy number (n = 102)	1.1 ± 0.6	1.2 ± 0.8	1.0 ± 0.5	0.116
86Y, % (n)	6.8 (7/103)	14.0 (7/50)	0 (0/53)	0.005
184F, % (n)	89.3 (92/103)	84.0 (42/50)	94.3 (50/53)	0.083
1034C, % (n)	11.7 (12/103)	20.0 (10/50)	3.8 (2/53)	0.011
1042D, % (n)	16.5 (17/103)	32.0 (16/50)	1.9 (1/53)	< 0.001
1246Y, % (n)	0 (0/103)	0 (0/50)	0 (0/53)	–
*cytb* 268S, % (n)	(0/50)	–	(0/50)	–
K13	52.5 (53/101)	18.4 (9/49)	84.6 (44/52)	< 0.001
G436S, % (n)	1.0 (1/101)	0 (0/49)	1.9 (1/52)	0.515
F483L, % (n)	2.0 (2/101)	2.0 (1/49)	1.9 (1/52)	0.737
Y493H, % (n)	1.0 (1/101)	0 (0/49)	1.9 (1/52)	0.515
G538 V, % (n)	1.0 (1/101)	0 (0/49)	1.9 (1/52)	0.515
R539T, % (n)	8.9 (9/101)	2.0 (1/49)	15.4 (8/52)	0.019
V568G, % (n)	1.0 (1/101)	2.0 (1/49)	0 (0/52)	0.485
C580Y, % (n)	38.6 (39/101)	12.2 (6/49)	63.5 (33/52)	< 0.001

Significant difference of mean *pfmdr* copy number between parasites collected before 2009 and from 2009 to 2016 determined by Independent *t* test

Significant differences of resistant alleles between parasites collected before 2009 and from 2009 to 2016 determined using Fisher’s Exact test

Single nucleotide polymorphisms (SNPs) on the *k13* gene of the parasite isolates were determined by PCR amplification and sequencing. The percentage of the *k13* mutation(s) was significantly higher among the parasites collected from 2009 to 2016 (44/52, 84.6%) compared with those collected before 2009 (9/49, 18.4%) (*p* < 0.001, Fisher’s Exact test). Seven different SNPs were identified including G436S, F483L, Y493H, G538V, R539T, V568G and C580Y (Table 3). The most common SNP found among these isolates were C580Y (38.6%). The *k13*-R539T allele, another common SNP in the Great Mekong Subregion, was identified in 8.9% of these isolates. Two mutations on the *k13* gene were identified in one isolate collected from 2009 to 2016 which were combinations of C580Y/F483L. The percentage of both C580Y and R539T had significantly increased (*p *= 0.019 and *p* < 0.001, respectively, Fisher’s Exact test) among the parasites collected from 2009 to 2016.

The parasite isolates were categorized in seven groups according to their genotype of the *pfmdr1* and K13 genes (Table 4), i.e., (I) the *pfmdr1* 86N allele with no mutation on the *k13* gene, (II) the *pfmdr1* 184F allele with no mutation on the *k13* gene, (III) the *pfmdr1* 184F allele with *k13* 580Y alleles (IV) the *pfmdr1* 184F allele with *k13* 539T alleles (V) the *pfmdr1* 184F + 1042D alleles with no mutation on the *k13* gene (VI) the *pfmdr1* 184F + 1034C + 1042D alleles with no mutation on the *k13* gene and (VII) others. Before 2009, the most prevalent parasites were those containing the *pfmdr1* 184F allele with no mutation on the *k13* gene (20/49, 40.8%). Parasites with the *pfmdr1* 86Y allele or the *pfmdr1* 184F + 1034C + 1042N alleles with no mutation on the *k13* gene were also identified before 2009, but not among the parasites collected from 2009 to 2016. Before 2009, the category VII contained one isolate with no mutation on both genes, with the *pfmdr1* 184F + 1042D alleles and *k13* 580Y alleles and with the *pfmdr1* 184F and *k13* 483L alleles were identified. Approximately 57% of parasites collected from 2009 to 2016 contained the *pfmdr1* 184F and *k13* 580Y alleles. In addition, 13% of these parasites exhibited the *pfmdr1* 184F and *k13* 539T alleles. In the group collected from 2009 to 2016, category VII consisted of one isolate with no mutation on both genes, two isolates with the *k13* 580Y allele but no mutation on the *pfmdr1* gene, one isolate with the *pfmdr1* 184F + 1034C and the *k13* 580Y alleles, one isolate with the *pfmdr1* 184F + 1034C and the K1 539T alleles. In addition, the *pfmdr1* 184F allele with the *k13* 436S, 493H and 538 V alleles was found in one isolate each.

**Table 4:** Haplotypes of *Plasmodium falciparum* isolates collected before 2009 and from 2009 to 2016

Group	Haplotype						Total	Before 2009	2009–2016
	*Pfmdr1*				K13		n (%)	n (%)	n (%)
	N86Y	Y184F	S1034C	N1042D	C580Y	R539T	100 (100.0)	49 (49.0)	51 (51.0)
I	86Y	Y184	S1034	N1042	C580	R539	7 (7.0)	7 (14.3)	0 (0)
II	N86	184F	S1034	N1042	C580	R539	26 (26.0)	20 (40.8)	6 (11.8)
III	N86	184F	S1034	N1042	580Y	R539	34 (34.0)	5 (10.2)	29 (56.9)
IV	N86	184F	S1034	N1042	C580	539T	8 (8.0)	1 (2.0)	7 (13.4)
V	N86	184F	S1034	1042D	C580	R539	5 (5.0)	4 (8.2)	1 (2.0)
VI	N86	184F	1034C	1042D	C580	R539	9 (9.0)	9 (18.4)	0 (0)
VII	Others						12 (12.0)	3 (6.1)	8 (15.7)

Significant differences of haplotypes between parasites collected before 2009 and from 2009 to 2016 determined using Chi square test, *p* < 0.001

### Correlation of anti-malarial drug sensitivities and genetic polymorphisms of *Plasmodium falciparum*

Table 5 shows in vitro anti-malarial sensitivities among the parasite isolates with different *pfmdr1* genotypes. The parasites containing the *pfmdr1* 86Y or 1034C or 1042D alleles exhibited significantly increased MQ sensitivity (*p* < 0.001, Independent *t* test) while, those containing the *pfmdr1* 184F allele showed significantly reduced MQ sensitivity (*p *= 0.043, Independent *t* test). The parasites with ≤ 1 copy of the *pfmdr1* gene showed higher MQ IC_50_ than those with more than one copy (*p* < 0.018, Independent *t* test). The parasites containing the *pfmdr*1 86Y allele exhibited significantly increased QN sensitivity compared with the wild-type counterpart (*p* = 0.008, Independent *t* test). The parasites with the *pfmdr1* 184F allele exhibited approximately twice less susceptible to QN than the parasites with the *pfmdr1* 184Y allele (*p *= 0.003, Independent *t* test). The parasites containing the *pfmdr1* N1042 allele or the *k13* 580Y alleles exhibited significantly higher PPQ IC_50_ compared with the others (*p* = 0.019 and *p* = 0.013, respectively, Independent *t* test). The parasites containing the *k13* 539T alleles exhibited significant higher ARS IC_50_ compared with the others (*p* = 0.017, Independent *t* test).

**Table 5:** Correlation of antimalarial drug sensitivities and *pfmdr1* and K13 gene of *P. falciparum* collected along the Thai–Cambodian border

Genotype	IC_50_ (nM)					
	CQ	QN	MQ	PPQ	ARN	DHA
*pfmdr1*						
86						
N86	107.1 ± 52.5	229.4 ± 130.6*	29.5 ± 26.0*	21.2 ± 10.9	3.0 ± 1.8	2.4 ± 1.1
86Y	67.8 ± 41.9	92.3 ± 71.0	7.6 ± 3.9	19.1 ± 7.7	2.5 ± 0.9	3.0 ± 1.8
184						
Y184	74.7 ± 38.3	104.1 ± 67.7*	12.6 ± 11.6*	19.9 ± 6.4	2.6 ± 1.1	2.9 ± 1.5
184F	108.2 ± 53.1	232.7 ± 131.2	29.6 ± 26.3	21.2 ± 11.1	3.0 ± 1.8	2.4 ± 1.1
1034						
S1034	103.3 ± 54.7	218.1 ± 135.8	30.6 ± 26.1*	21.6 ± 10.9	3.0 ± 1.8	2.4 ± 1.1
1034C	116.2 ± 33.1	231.2 ± 103.8	8.4 ± 7.8	10.9 ± 7.8	2.6 ± 1.5	2.6 ± 1.5
1042						
N1042	104.2 ± 55.8	221.2 ± 133.0	31.9 ± 26.4*	22.2 ± 10.8*	3.0 ± 1.8	2.4 ± 1.1
1042D	107.8 ± 35.0	260.5 ± 122.2	8.7 ± 6.2	14.4 ± 7.2	2.8 ± 1.6	2.7 ± 1.3
Copy no.						
≤ 1	104.8 ± 49.4	227.8 ± 140.0	33.9 ± 30.2*	18.8 ± 6.3	3.2 ± 1.7	2.5 ± 1.2
> 1	105.9 ± 56.4	214.3 ± 123.0	21.9 ± 18.0	22.9 ± 13.2	2.9 ± 1.8	2.5 ± 1.1
K13						
539						
R539	106.3 ± 54.7	212.0 ± 125.7	26.7 ± 24.5	21.6 ± 10.9	2.9 ± 1.7	2.5 ± 1.2
539T	96.2 ± 38.9	272.7 ± 189.2	42.1 ± 34.5	16.4 ± 10.0	4.3 ± 1.8*	2.0 ± 0.7
580						
C580	97.6 ± 39.9	216.5 ± 128.8	27.3 ± 26.5	18.1 ± 8.7	2.7 ± 1.4	2.6 ± 1.2
580Y	118.9 ± 69.6	219.3 ± 140.8	29.6 ± 24.6	25.5 ± 11.9*	3.4 ± 2.1	2.3 ± 1.1

* Represents a significant difference of mean IC_50_ ± SD at *p* < 0.05, Independent *t* test

Table 6 shows the comparisons of in vitro anti-malarial sensitivities of *P. falciparum* isolates with different *pfmdr1* and *k13* genotypes. Only six groups (I–VI) were compared because varied genotypes with a small number were added in group VII. Significant differences were observed in the MQ and PPQ IC_50_s among these six groups (*p* = 0.002 and 0.013, respectively, One-way ANOVA). Post Hoc analysis using Scheffe test showed no significant difference between any pair of these genotypes which may have been due to a lower number of parasites in some subgroups.

**Table 6:** In vitro antimalarial sensitivities of different haplotype subgroups of *P. falciparum*

Drug IC_50_ (nM)	Haplotypes						*p* value
	I (n = 7)	II (n = 26)	III (n = 34)	IV (n = 8)	V (n = 5)	VI (n = 9)	
CQ	67.8 ± 41.9	93.8 ± 37.9	118.8 ± 71.0	99.6 ± 40.3	97.6 ± 43.6	120.4 ± 30.9	0.188
QN	92.3 ± 71.0	207.6 ± 100.7	222.2 ± 142.5	290.7 ± 193.9	281.3 ± 155.4	259.5 ± 98.7	0.053
MQ	7.6 ± 3.9	36.8 ± 27.0	31.1 ± 25.6	43.5 ± 36.6	12.7 ± 7.6	6.1 ± 3.1	0.002
PPQ	19.1 ± 7.7	17.2 ± 7.3	25.6 ± 12.3	16.8 ± 7.4	11.0 ± 4.3	16.3 ± 8.0	0.013
ARS	2.5 ± 0.9	2.3 ± 1.0	3.4 ± 2.2	4.2 ± 1.9	3.1 ± 1.7	1.5 ± 0.5	0.063
DHA	3.0 ± 1.8	2.6 ± 1.1	2.3 ± 1.2	2.1 ± 0.6	2.6 ± 0.8	2.8 ± 1.6	0.588

Significant difference of mean IC_50_ was determined by One-way ANOVA

## Discussion

Due to the emergence of artemisinin resistance along the Thai–Cambodia border especially in Chanthaburi and Trat Provinces, the artemisinin resistance containment project was launched in 2009 by the Ministry of Public Health, Thailand [17]. Reduction of artemisinin pressure was one of objectives in this project by replacing AS–MQ with a fixed dose combination of ATQ–PG. In this study, both phenotypes and genotypes of *P. falciparum* isolates collected after the artemisinin resistance containment project, 2009–2016 were compared with the isolates collected before 2009. The parasites collected from 2009 to 2016 showed significantly higher CQ, QN, MQ, PPQ and AS IC_50_s compared with the parasites collected before 2009. The IC_50_s of these Thai isolates were in the same range as those reported in Cambodian isolates in 2013 [33]. Previously described cut-off points for in vitro anti-malarial resistance were used to determine the parasite’s resistant phenotypes [28–32]. Of 101, only one isolate collected before 2009 exhibited CQ sensitive. No CQ-sensitive isolate was detected among the parasites collected from 2009 to 2016. Although CQ was not used to treat falciparum malaria, Thai isolates of *P. falciparum* remain resistant to CQ. This may be due to vivax malaria sharing similar endemic areas with falciparum malaria. Thus, CQ, the first-line treatment for vivax malaria could cause a drug pressure for *P. falciparum* as well. The cure rate of MQ has rapidly declined soon after using as a monotherapy to treat falciparum malaria in 1991 [34]. Because MQ is a long half-life drug, drug pressure could cause the emergence of MQ resistance. As a result, AS–MQ combination was used as the first-line treatment of uncomplicated falciparum malaria since 1995. The parasites collected from 2009 to 2016 showed significantly increased MQ IC_50_ compared with the parasites before 2009. In addition, approximately 57% of the isolates collected after 2009 exhibited in vitro MQ resistance. Increased MQ resistance after 2009 may be influenced by the delayed parasite clearance phenotype of *P. falciparum* against AS in these areas. Slow parasite clearance causes more parasites to be exposed to the partner drug, i.e., MQ, increasing the risk of resistance selection of the partner drug, which in turn increases the risk of treatment failure. In the present study, the parasites with the *pfmdr1* 184F allele showed a significantly higher MQ IC_50_ than others.

A few cases of ATQ–PG treatment failure have been reported. Treatment failure of ATQ–PG was due to ATQ resistance and has been linked to point mutations in the target gene, the *cytb* gene [20, 32, 35]. Determination of the phenotypes and genotypes related to ATQ–PG response in Thai isolates of *P. falciparum* will be useful for rational drug use. According to Musset and colleagues (2006) [32], the cut-off point for in vitro ATQ resistance was the IC_50_ > 1900 nM. None of parasite isolates in this study exhibited ATQ resistance. In addition, they contained no mutations in the *cytb* 268 codon, molecular markers for ATQ resistance. The present results are similar to our survey in 2008 showing no evidence of ATQ resistance in Thai isolates of *P. falciparum* collected from both Thai–Cambodian and Thai–Myanmar borders [36]. A recent study in Cambodia also showed similar results indicating that *P. falciparum* isolated from Western Cambodia remained sensitive to ATQ in vitro and showed no point mutations in the *cytb* gene [33]. Recently, a successful cure of a multidrug-resistant *falciparum* case after artemisinin-based and QN-based treatment failure was reported in a subject that traveled to Cambodia [37]. These results suggest that a fixed-dose combination of ATQ–PG could be used the artemisinin-resistant areas with careful monitoring.

In this study, the IC_50_ of AS but not DHA was increased among the parasites isolated from 2009 to 2016. However, using the IC_50_ of > 10.5 nM as the cut-off point for in vitro AS and DHA resistance [31], no parasite exhibiting AS and DHA resistance was collected in the year before and after the artemisinin resistance containment project. Although no evidence exists of full artemisinin resistance, partial artemisinin resistance defined by delayed parasite clearance following treatment with an AS monotherapy or with an ACT is widespread in the Great Mekong Subregion [38]. To date, more than 200 nonsynonymous mutations in the *k13* gene have been reported. Several mutations in the *k13* gene were associated with delayed parasite clearance in vivo and in vitro including N458Y, Y493H, R539T, I543T, R561H and C580Y [38]. In the GMS, mutations in the *k13* gene have spread and are distinctly reported according to their geographical areas [38, 39]. In the eastern GMS including Thai–Cambodia border, C580Y, R539T, Y493H, I543T, and P553L were commonly identified with the domination of C580Y. In the present study, 7 SNPs were identified including G436S, F483L, Y493H, G538 V, R539T, V568G, and C580Y. The most common SNPs of the isolates collected from 2009 to 2016 were C580Y (63.5%) and R539T (15.4%). Ring survival assay was not performed in the present study, however, reduced in vitro AS sensitivity was identified by in vitro sensitivity assay in the parasites with the *k13* 539T allele compared with others. For the newly identified SNPs including G436S and F483L, validation as a resistance marker will be required.

After 2009, ATQ–PG has been used to reduce artemisinin pressure in this area. In the absence of drug pressure, some resistant parasites might be less fit than their sensitive counterparts [40]. However, after the artemisinin resistance containment project, *k13* mutations had increased significantly from 18.4 to 84.6%. Of these mutations, the *k13* C580Y allele is increasing and replacing other haplotypes along the Thai-Cambodia border indicating a selective sweep in these areas. A study of parasites collected in 2007 found that 50% (11/22) of parasites from Chanthaburi and Trat Provinces contained the *k13* mutations [41]. Of these, 45.5% (10/22) contained the *k13* 580Y allele indicating that parasites with the *k13* 580Y allele spread widely before the artemisinin resistance containment project. Recent studies indicated that the parasites with the *k13* 580Y allele arose in western Cambodia and then spread to other countries in the western Great Mekong Subregion including Thailand, Lao PDR and Vietnam [12, 41–43]. Thus, sensitive parasites might not compete with the main haplotype, the *k13* 580Y allele. One other factor that might influence the spread of artemisinin resistance in these areas is cross-board migration. Approximately one half of malaria cases in Thailand were foreign migrant workers [2]. As part of the artemisinin resistance containment project in Thailand, AS–MQ has been replaced by ATQ–PG to reduce artemisinin pressure in Chanthaburi and Trat provinces, Thailand which has been implemented since 2009. However, ACT remains the first-line treatment for uncomplicated falciparum malaria in Cambodia [38]. Thus, artemisinin pressure in these areas might not be effectively reduced. In addition, different policies and implementation of primaquine, as a *P. falciparum* gametocytocide, may influence the spreading of artemisinin resistance along Thai–Cambodian border [44, 45].

DHA-PPQ was used as the first-line drug for multidrug-resistant falciparum malaria in Cambodia [46]. Unfortunately, treatment failure of DHA-PPQ was promptly reported possibly due to the existing resistant parasites because PPQ monotherapy was used in Cambodia in the 1990s [47]. A few studies showed a link between the *pfmdr1* copy number and PPQ sensitivity [48–50]. However, no significant difference of PPQ IC_50_ between the parasites with one and more than *pfmdr1* copy number was found in the present study (18.8 ± 6.3 and 22.9 ± 13.3, *p* = 0.078, Independent *t* test) similar to the recent study using parasites collected from both Thai–Myanmar and Thai–Cambodian border areas [51]. Genetic markers for PPQ resistance including nonsynonymous SNP encoding a Glu415Gly mutation in a putative exonuclease (exo-E415G) and plasmepsin 2–3 amplification have been identified [52, 53]. Both in vitro PPQ sensitivity and ring survival assay were used to identify the association between PPQ resistance and these genes. Treatment failure of DHA-PPQ in Cambodia has been associated with parasites containing the *k13* mutations and multiple plasmepsin 2 copy [53]. Recent studies have shown that parasites with the *k13* 580Y allele and plasmepsin 2 amplification have emerged and spread widely in the western Mekong Basin Subregion causing DHA-PPQ treatment failure [42, 43]. In the present study, the parasites containing the *k13* 580Y alleles exhibited significant higher PPQ IC_50_ compared with the others. This could be explained by parasites with the 580Y allele acquiring reduced PPQ susceptibility in this area. Unfortunately, other genetic markers for PPQ resistance including exo-E415G and plasmepsin 2–3 amplification were not determined in our study.

## Conclusion

ATQ–PG, one of the non-ACT combinations, might be suitable to treat uncomplicated falciparum malaria in multidrug-resistant areas. Reduced ATQ sensitivity and mutation in the target gene has not been identified after the artemisinin resistance containment project. However, reduced artemisinin pressure using this combination might not be accomplished. Parasites with the *k13* mutations, particularly the C580Y mutation, have increased over the years, even after the artemisinin resistance containment project. In addition, reduced sensitivity of the partner drugs of ACT including MQ and PPQ has been shown. New combinations which overcome these resistant phenotypes and genotypes should be carefully selected.
